# Long-Term Clinical Outcome and Prognosis After Thrombectomy in Patients With Concomitant Malignancy

**DOI:** 10.3389/fneur.2020.572589

**Published:** 2020-10-15

**Authors:** Sogo Oki, Masahito Kawabori, Sumire Echizenya, Yusuke Shimoda, Daisuke Shimbo, Toshiya Osanai, Kazuki Uchida, Kiyohiro Houkin

**Affiliations:** ^1^Department of Neurosurgery, Hokkaido University Graduate School of Medicine, Sapporo, Japan; ^2^Department of Neurosurgery, Hokkaido Medical Center, Sapporo, Japan; ^3^Department of Neurosurgery, Teine Keijinkai Hospital, Sapporo, Japan

**Keywords:** thrombectomy, malignancy, cancer, large vessel occlusion, Trousseau syndrome, prognosis

## Abstract

Endovascular thrombectomy (EVT) is the preferred treatment strategy for patients with acute ischemic stroke (AIS). However, clinical outcome and prognosis in patients who undergo EVT in response to AIS with concomitant malignancy have not been fully elucidated. Data of patients with malignancy who underwent EVT at participating institutions between January 2015 and April 2019 were retrospectively analyzed. Patient characteristics, treatment methods, posttreatment strategy, and long-term prognosis were evaluated in 12 patients with prediagnoses of malignancy. Good revascularization (TICI 2b or higher) was achieved in 10 of 12 patients. Among the eight patients who survived more than 2 weeks from onset, four patients showed good clinical outcome [modified Rankin Scale (mRS) <2] at 60 days posttreatment and were able to continue treatment for malignancy. However, seven of eight patients died within a year of EVT (median survival, 83 days) due to progression of malignancy. One-year survival was achieved in only one patient whose etiology of stroke was determined as infectious endocarditis and not Trousseau syndrome. Even after successful revascularization and good short-term clinical outcome, the long-term prognosis after thrombectomy in patients with malignancy was poor. Thrombectomy for concomitant malignancy requires judicious decision, and further studies are necessary to fully elucidate its efficacy.

## Introduction

Stroke is a well-known comorbidity in patients suffering from malignancy, and 5–15% of these patients experience cerebrovascular thromboembolic events during their clinical course (1, 2). The risk of short-term mortality is higher in patients of ischemic stroke with malignancy than in those without concomitant malignancy (3), presumably because stroke leads to lower performance status, which subsequently results in ceasing the treatment of malignancy. The treatment of stroke in patients with malignancy is challenging because of the diversity in the underlying mechanisms that cause stroke, including hypercoagulability, atherosclerosis, and noncardiac origin embolisms (4, 5).

The development of endovascular thrombectomy (EVT) is a recent advancement, and numerous randomized clinical trials have proved the efficacy of EVT for the treatment of acute ischemic stroke (AIS) in patients with large vessel occlusion (LVO) (6–11). However, the presence of terminal illness or short life expectancy was an exclusion criterion in many of these trials. Therefore, the clinical results of EVT in patients with concomitant malignancy have not been fully elucidated. Short-term results of EVT in concomitant cancer patients seem favorable (12–14). However, long-term prognosis has not been reported. In this study, we assessed the clinical features of patients who underwent EVT for AIS due to concomitant malignancy and their clinical course with a special focus on long-term prognosis.

## Materials and Methods

### Study Population

This study was approved by the institutional review boards of the participating institutes, and all the patients or their families provided written informed consent. A retrospective review of data, between April 2015 and March 2019, using our local stroke database was conducted (15). Concomitant malignancy was defined when the patient was diagnosed in the past 6 months, when the patient was undergoing malignancy treatment, or when the patient showed progression, recurrence, or metastasis of malignancy (4, 16). Patient information, including age, sex, location of malignancy, cell type, cancer staging, malignancy treatment before stroke onset, concomitant stroke risk factors (hypertension, diabetes, hyperlipidemia, atrial fibrillation, pulmonary embolism or deep venous thrombosis, and previous history of stroke), premorbid antiplatelet/anticoagulant drug, and blood D-dimer level at admission (17) were obtained from the medical records.

### Treatment Strategy

Treatment strategies for EVT indication and associated procedures have been previously reported (15). Briefly, National Institutes of Health Stroke Scale (NIHSS) and prestroke modified Rankin Scale (mRS) were used by neurologists upon arrival of the patient at the Emergency Department for neurological assessment. Nonenhanced cranial computed tomography (CT) scan and/or magnetic resonance (MR) imaging including diffusion-weighted imaging (DWI) and magnetic resonance arteriography (MRA) were obtained as soon as possible to confirm diagnosis (18). Once the patient was diagnosed with AIS of LVO, the Alberta Stroke Program Early CT score (ASPECTS) or MRI score was used to measure the ischemic lesion size (19), and the efficacy of treatment with EVT was decided upon by stroke neurologists and neurosurgeons. The will of patient and caregivers was also considered for decision making. Intravenous recombinant tissue plasminogen activator (rtPA) was used within 4.5 h after symptom onset if the patients met the relevant criteria. EVT was performed using stent retrievers (Solitaire FR, ev3 Inc., Irvine, CA, USA; Trevo, Concentric Medical Inc., Mountain View, CA, USA; Revive SE, Raynham, MA, USA; or Codman Neuro/DePuy Synthes, Johnson and Johnson) or forced suction system (Penumbra 5 MAXACE, Penumbra Inc., Alameda, CA, USA) selected according to the preference of the surgeon. Angiographical reperfusion was evaluated by thrombolysis in cerebral infarction score (TICI) (20), and successful reperfusion was defined as a score >2b. Time from onset to recanalization (O2R) and groin puncture to recanalization (P2R) was also examined. After the EVT procedure, patients were transferred to the stroke care unit, and follow-up MR imaging and angiography were performed on the same day or on the following day to ensure the patency of the treated vessel and to check for any periprocedural complications.

### Clinical Outcome

The clinical course of the patient was obtained from the medical records or by telephone interview. The clinical outcome measurements were assessed by mRS score at 14, 30, 60, and 90 days, as well as during the poststroke malignancy treatment. The date and the reason of mortality were also recorded when the patient died within a year of the EVT.

### Statistics

Continuous variables are summarized as mean ± standard deviation or median with interquartile range.

## Results

**Figure 1:**
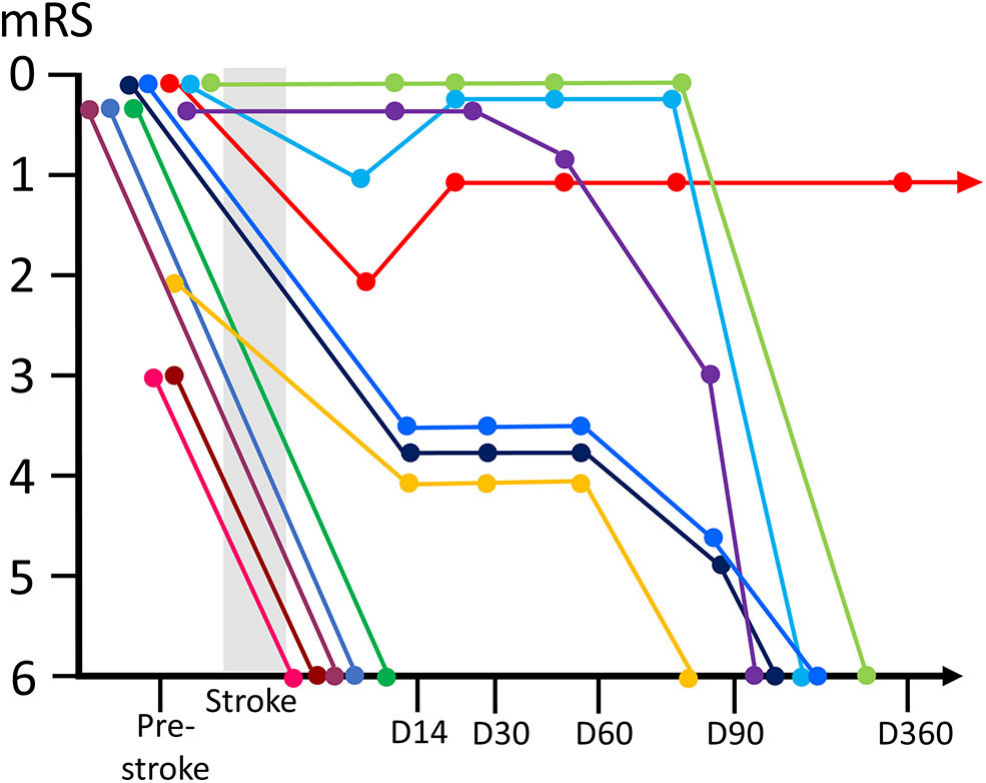
Time courses of the clinical outcome (modified Rankin Scale; mRS) and prognosis of malignancy patients with EVT. Clinical outcome trajectories (from pre-stroke period till death) for individual patients are represented using different colors. EVT, endovascular thrombectomy.

Thrombectomy for patients with concomitant malignancy was found in three participating hospitals, and a total of 124 cases of thrombectomy were performed during this period (Hokkaido University Hospital; 9 cases, Hokkaido Medical Center; 41 cases, Teine Keijinkai Hospital 74 cases). Among them, data of 12 patients (9.6%) with concomitant malignancy who underwent EVT for AIS were consecutively collected from the stroke data base. Clinical features of the patients are provided in Table 1. The mean age of the patients was 64.0 ± 14.5 years. Ten patients were undergoing malignancy treatment, and two patients were newly diagnosed at the time of stroke onset. Of the 12 patients, 50% of the patients (*n* = 6) had lung cancer, followed by uterine (25%; *n* = 3), ovarian (16.7%; *n* = 2), and colon (8.3%; *n* = 1) cancer. Seven patients (58.3%) showed advanced malignancy (cancer stage IV) or recurrent cancer, and adenocarcinoma was found in eight cases (66.7%). Atrial fibrillation was found in only one patient. Patients did not show an obvious increase in the trend of the presence of vascular risk factors (hypertension, diabetes, and hyperlipidemia). Six patients were prescribed with anticoagulant drug [warfarin or direct oral anticoagulant (DOAC)], and one patient was prescribed with antiplatelet drug before the onset of stroke. The reasons for prescription of anticoagulant were pulmonary embolism/deep venous thrombosis (*n* = 3), previous history of stroke (Trousseau syndrome; *n* = 2), and stroke prophylaxis of atrial fibrillation. Blood test revealed high D-dimer values at admission ranging from 3.7 to 64.9 (mean, 23.8 ± 18.3) g/ml. Prestroke mRS score was 0 for nine patients, 2 for one patient, and 3 for two patients. NIHSS score at admission was 17.2 [interquartile range (IQR), 14–21] and ASPECTS ranged from 8 to 10. The location of atrial occlusions was determined at the internal cerebral artery (ICA) (*n* = 2), proximal portion of the middle cerebral artery (MCA) (M1; *n* = 8), and middle portion of the MCA (M2; *n* = 2). Treatment and prognostic results are shown in Table 2. Four of 12 patients (33.3%) received IV t-PA before or during EVT procedure. Retrievable stents were used in five patients, forced suction devices were used in four patients, and both devices were used in three patients. Median O2R was 264 min (IQR, 179–318 min), and median P2R was 52 min (IQR, 38–101 min). Successful reperfusion was achieved in 10 of the 12 patients and TICI 2a in two patients. Three patients showed hemorrhagic infarction, and one patient showed subarachnoid hemorrhage as symptomatic complication. Reocclusion of the affected artery was found in three patients, all of which were found by nearest follow-up MRA (two on the following day and one at 6 days from thrombectomy), and the lesions were the same with the prior occluded ones. Five patients died within 2 weeks due to stroke sequelae. Among those who survived for more than 2 weeks (*n* = 7), five patients were able to continue malignancy treatment including surgery, chemotherapy, and radiotherapy. However, two patients were given best supportive care because of their low performance status. Four patients regained functional independence (mRS ≤ 2) at 2 months from the onset of stroke, but three of them died within a year. Overall, 11 of 12 patients died because of malignancy progression or stroke sequelae within a year (Figure 1). One-year survival was achieved in only one patient. Poststroke examination in this patient confirmed the etiology of stroke to be infectious endocarditis and not Trousseau syndrome. The patient is still alive at the time of publication (>2.5 years).

**Table 1:** Patient characteristics.

**Case no**.	**Age/sex**	**Malignancy type/stage**	**Cancer stage**	**Malignancy treatment before stroke onset**	**HT**	**DM**	**HL**	**Af**	**PE/DVT**	**Prior stroke**	**Prestroke antithrombotic medication**	**D-Dimer (μg/ml) at stroke onset**	**NIHSS on admission**	**Prestroke mRS**	**ASPECTS**	**Site of occlusion**
1	75/M	Lung squamous carcinoma	III	Chemo-Radiotherapy	+	–	–	–	–	–	None	29.8	20	0	11 (MRI)	Lt.ICA (C2)
2	40/F	Ovarian clear cell adenocarcinoma	III	Surgery	–	–	–	–	+	–	Edoxaban	21.0	18	0	8 (MRI)	Rt.M1
3	68/F	Colon adenocarcinoma	IV	Surgery, chemotherapy	–	–	–	–	–	–	None	35.9	18	2	8 (MRI)	Rt.M1
4	61/F	Uterine body cancer	IV	Surgery, chemoradiotherapy	–	–	–	–	–	+	Edoxaban	21.3	15	0	10 (CT)	Rt.ICA (C1)
5	70/M	Lung cancer	IV	None	–	–	–	–	–	–	None	64.9	10	0	9 (MRI)	Rt.M1
6	72/F	Ovarian adenocarcinoma	IV	Surgery, chemotherapy	–	–	–	–	–	–	None	18.9	14	0	9 (MRI)	Lt.M1
7	67/F	Lung adenocarcinoma	IV	Chemotherapy	–	–	+	–	–	+	None	3.1	21	0	8 (MRI)	Lt.M1
8	36/F	Uterine cervical adenocarcinoma	Recurrence	Surgery, chemoradiotherapy	–	–	–	–	+	-	Edoxaban	7.7	8	0	9 (MRI)	Rt.M1
9	74/M	Lung small cell carcinoma	III	None	–	–	–	–	–	–	Aspirin	14.7	25	3	8 (CT)	Lt.M1
10	74/M	Lung adenocarcinoma	Recurrence	Surgery, chemoradiotherapy	+	–	+	–	–	–	Edoxaban	47.1	22	3	8 (MRI)	Lt.M1
11	49/F	Uterine cervical adenocarcinoma	III	Chemoradiotherapy	+	–	–	–	+	–	Edoxaban	17.7	11	0	10 (MRI)	Rt.M2
12	78/M	Liver hepatic cell carcinoma	III	Chemotherapy	–	+	–	+	–	–	Warfarin	3.7	30	0	10 (CT)	Lt.M2

**Table 2:** Treatment results.

**t-PA**	**TICI**	**O2R (min)**	**P2R (min)**	**Successful recanalization on the following day**	**Symptomatic complication**	**mRS**				**Poststroke treatment**	**Overall survival**
						**D14**	**D30**	**D60**	**D90**		
–	3	161	31	+	–	6	6	6	6	BSC	4 days
–	2b	203	38	+	–	2	1	1	1	Surgery, chemotherapy	Present (>2.5 years)
**+**	2b	179	52	–	–	4	4	4	6	BSC	85 days
–	3	121	21	+	–	0	0	0	0	Radiotherapy	355 days
**+**	3	365	101	–	–	6	6	6	6	BSC	12 days
–	2b	451	51	+	–	1	0	0	0	Chemotherapy	150 days
–	2a	285	55	–	–	4	4	4	5	BSC	140 days
–	3	161	46	+	–	0	0	1	3	Chemotherapy	121 days
**+**	2a	318	30	N/A	HI	6	6	6	6	BSC	1 days
–	2b	197	130	N/A	SAH	6	6	6	6	BSC	0 day
**+**	2b	266	116	+	HI	4	4	6	6	Chemotherapy	56 days
–	2b	462	177	+	HI	6	6	6	6	BSC	6 days

*BSC, best supportive care; min, minutes; HI, hemorrhagic infarction; N/A, not applicable; O2R, onset to reperfusion; SAH, subarachnoid hemorrhage; TICI, Thrombolysis in Cerebral Infarction score; t-PA, tissue plasminogen activator; +, present or done; –, not present or not done*.

### Representative Case (Case 8)

**Figure 2:**
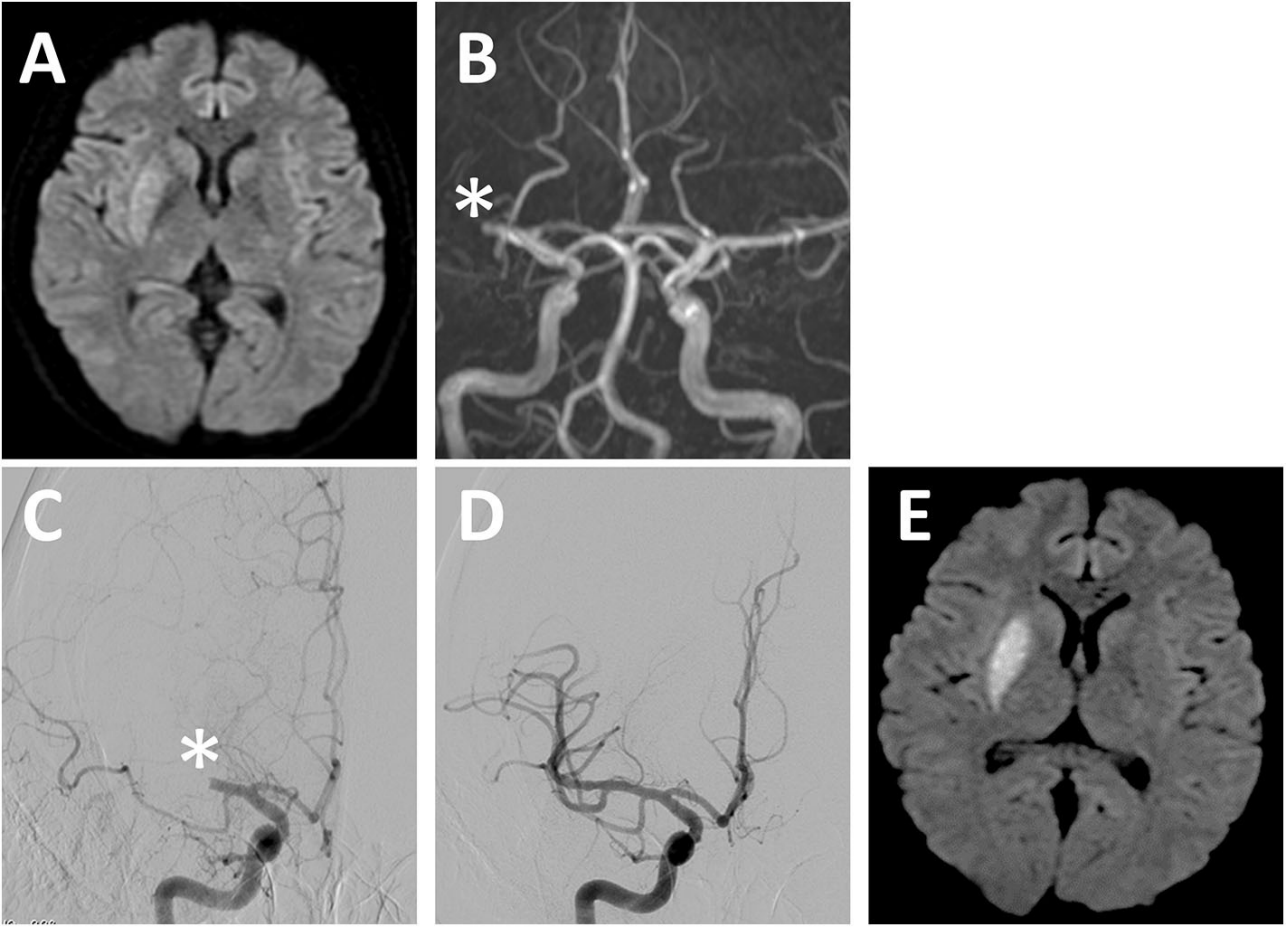
Representative case. **(A)** DWI and **(B)** MR angiography image on admission. MR angiography images at **(C)** pretreatment and **(D)** posttreatment with EVT. DWI on the following day of EVT **(E)**. EVT, endovascular thrombectomy; DWI, diffusion-weighted imaging; MR, magnetic resonance. *Occluded vessel.

The patient was a 36-year-old woman who underwent extended hysterectomy and chemotherapy for stage IIb uterine cervical cancer (mucinous adenocarcinoma) 1 year before the onset of stroke. Recurrence of the cancer was diagnosed with contrast-enhanced computed tomography (CT) that showed metastasis to para-aortic lymph node 4 months before the onset of stroke after which the patient started chemoradiotherapy. Edoxaban (60 mg daily) was started 1 month before the stroke because the patient was diagnosed with right subclavian venous thrombi, presumably related to central venous port. The patient was transferred to our hospital due to sudden left hemiparesis. Their NIHSS score was 8, and blood examination showed an elevated concentration of D-dimer (7.7 μg/ml). MR imaging showed occlusion of right M1 with ASPECTS of 8 (Figures 2A,B). t-PA was not administered because of the low platelets counts (<100,000/μl), and EVT was decided based on the timing of stroke (i.e., <8 h), neurological severity, and imaging data. Combination of a stent-retrieval device (Solitaire) and an aspiration device (Penumbra 5MAX ACE) was selected. White thrombi were successfully removed by first pass (TICI3) (Figures 2C,D), and the O2R was 161 min. Pathological examination of the thrombus revealed that it mostly consisted of fibrin, and no tumor cells were found. The MR imaging taken on the following day showed minimal ischemic lesion (Figure 2E). Further, the neurological symptoms of the patient showed dramatic improvement (NIHSS score 1). The patient was discharged on day 10 with no neurological deficit. Edoxaban was replaced with subcutaneous heparin calcium after consulting with the patient, and the patient did not experience stroke recurrence thereafter. Chemotherapy was resumed; however, it was difficult to control the progression of cancer. The patient died from respiratory failure due to malignant pleural effusion on postoperative day 121.

## Discussion

In this retrospective study, we elucidated several new aspects of EVT in patients with concomitant malignancy. First, even with successful revascularization and good short-term prognosis, patients with malignancy showed unfavorable long-term prognosis (11 out of 12 patients died within a year). Second, anticoagulants do not seem to be enough to block the occurrence of ischemic stroke in patients with malignancy, as in this study, half of the patients were taking DOAC or warfarin before the ischemic onset. Further, approximately one-third of the patients experienced reocclusion of the affected artery on the day following the EVT procedure, presumably because of abnormal hemostasis in patients with malignancy.

Even with a high rate of recanalization (83%), we found that short-term functional independence (mRS, 0–2) was achieved in only 25% of the patients in this study, which seems to be lower than that observed in previously reported clinical trials (33–72%) (6–10). This incongruity can be attributed to the fact that the treatment criteria in many of the patients in this study were different from those used in the randomized trial designs. Moreover, our result was similar to the comprehensive results of our previously reported EVT trial (25%), which is considered as real world data (15). However, short-term mortality rate in this report (50%) was higher than that observed in clinical trials (8–21%) as well as our previous report (12%). This is probably because ischemic stroke frequently occurs at the late stage of malignancy when the condition of the patients is not as good as that of the participants in clinical trials (3). Short-term prognosis for patients who underwent EVT with concomitant malignancy has been reported by others, and the results were mostly similar (Table 3) (21, 22). Jung et al. showed the result of EVT for 19 patients with active cancer and reported that only three patients (16%) were able to attain functional independence, while the rest were either bedridden (4 patients; 21%) or died (12 patients; 63%) (21). Sallustio et al. showed that functional independence was achieved in 42% of the patients, while they noticed a mortality rate of 29% (22). They also reported that no difference was found in successful reperfusion, 3-month functional independence, and symptomatic intracranial hemorrhage. However, the mortality after 3 months was more than double in patients with active cancer compared with that in controls (29.1 vs. 12.5%). Further, they concluded that active cancer *per se* was an independent predictor of a decrease in functional independence at 3 months (23). Both these reports did not show long-term (>12 months) prognosis in the patients of stroke who required EVT for AIS. Thus, to the best of our knowledge, this is the first report to reveal poor long-term prognosis after EVT in patients with malignancy, in which case 92% of the patients died within a year. Even though thromboembolism is the second leading cause of death for patients with cancer (24), the mortality in our study is higher than that expected in patients with malignancy. This is because the onset of stroke is considered to affect the overall mortality in patients with malignancy due to the contribution of various factors including termination of malignancy treatment and worsening of general condition (immune system, nutrient state, and frailty). However, our results showed that even with the poor long-term prognosis, EVT could at least recover performance status and enable improved quality of life in some patients (14).

**Table 3:** Previous results of thrombectomy for stroke patient with malignancy.

**Authors**	**Jung et al. (21)**	**Sallustio et al. (22)**	**Present report**
Number of cases	19	24	12
Age (years, mean/median)	69 (median)	69 (mean)	64 (mean)
Baseline NIHSS (mean/median)	16 (median)	14.2 (mean)	17.2 (mean)
Cancer stage IV (%)	89%	42%	58%
Prestroke mRS ≦ 2 (%)	N/A	96%	83%
IV thrombolysis (%)	17%	50%	33%
P2R (min)	30	53	70
TICI 2b or 3 (%)	37%	77%	83%
mRS 0–2 at 3 months (%)	16%	42%	25%
mRS 6 at 3 months (%)	63%	29%	50%
mRS 0–2 at 12 months (%)	N/A	N/A	8%
mRS 6 at 12 months (%)	N/A	N/A	92%

*IV, intravenous; mRS, modified Rankin Scale; P2R, puncture to reperfusion; N/A, not applicable*.

Further, we found that of the six patients (50%) who were taking anticoagulant medication at the time of stroke onset. We speculate that this was because of the hypercoagulation status of the patients with malignancy. Warfarin or DOAC (with preference for edoxaban and ribaroxaban) is recommended in several guidelines for the prevention of venous thrombosis and stroke in patients with active malignancy (25–27), yet it is quite difficult to completely avoid the occurrence of stroke in these patients. Further, progressive or stage IV malignancy has been reported to show higher risk of thromboembolic event (2, 28, 29). Therefore, better understanding of the pathology of stroke in case of active malignancy is highly warranted to design paradigms for effective prophylaxis.

We also experienced three patients whose affected arteries showed reocclusion after successful recanalization, which is not often the case with cardioembolic stroke. All of these patients showed complete clearance of the thrombus and had no atherosclerotic change at the affected artery. Activated endothelial cells at the affected artery that excessively express adhesion molecules and results in blood–brain barrier damage concomitant with systemic hypercoagulation status of the patients may account for the reocclusion (30, 31). This is also reported in the recent COVID-19 patients in which 40% of the successful revascularized patients showed early cerebral reocclusion (32). Further study of this phenomenon is highly warranted.

This study has several limitations. First, it was a retrospective study with data from our affiliated hospitals, and the sample size was too small to draw valid conclusions for outcome of EVT in patient with concomitant malignancy. Larger cohorts would be necessary to elucidate the effect of EVT in patients with malignancy. Second, there may have been a bias in selecting EVT candidates. This was because our records only contain information on patients who underwent EVT, and the cases in which the patient refused to be treated or EVT was not decided by the neurologist could not be followed. Prospective trials or matched cohorts would be needed to show the implication of our result.

## Conclusion

Here, we report the outcome of EVT in AIS patients with concomitant malignancy. Even with a high rate of recanalization and satisfactory short-term result, the long-term prognosis was surprisingly poor. However, the patient who had a clear etiology of stroke (infectious endocarditis) showed long-term survival, and those who gained functional recovery benefit their quality of life at least for some duration. Further examination and better understanding of the pathophysiology of this disease are necessary to establish better treatment strategies.

## Data Availability Statement

The raw data supporting the conclusions of this article will be made available by the authors, without undue reservation.

## Ethics Statement

The studies involving human participants were reviewed and approved by the institutional review boards, Hokkaido university hospital, division of clinical research administration. The patients/participants provided their written informed consent to participate in this study.

## Author Contributions

SO and MK: conceptualization. MK: methodology, writing—review and editing, supervision, and funding acquisition. SE, DS, TO, KU, and KH: investigation. SO: writing—original draft preparation. All authors have read and agreed to the published version of the manuscript.

### Conflict of Interest

The authors declare that the research was conducted in the absence of any commercial or financial relationships that could be construed as a potential conflict of interest.
